# Complete response to nivolumab in *Kirsten rat sarcoma virus* oncogene *KRAS*-G12C mutant metastatic lung adenocarcinoma: a case report

**DOI:** 10.1186/s13256-022-03593-3

**Published:** 2022-11-03

**Authors:** Jeffrey Mathew Boby, Nurul Nadia Mohd Ghazali, Anita Mani, Mathew George

**Affiliations:** 1 Government Medical College, Kozhikode, India; 2 Calvary Mater Newcastle, Newcastle, Australia; 3 Pathology North, Tamworth Hospital, Tamworth, Australia; 4 North West Cancer Centre, Tamworth, Australia

**Keywords:** Non-small cell lung cancer, Metastatic, *KRAS* G12C mutant, Immunotherapy, Nivolumab, Complete response

## Abstract

**Background:**

The advent of immunotherapies has ushered in a new era in the treatment of non-small cell lung carcinoma. Although immunotherapies are associated with improved clinical outcomes, studies report a median overall survival of 11 months with progression-free survival of 2.5 months with the use of nivolumab for pretreated metastatic non-small cell lung cancer. Herein, we describe a case of advanced non-small cell lung carcinoma that has shown exceptional response to immunotherapy, with the patient being in complete response for the past 6 years since commencement of nivolumab.

**Case presentation:**

We report the case of a 58-year-old female Caucasian, an ex-smoker with 40-pack-year history of smoking, who presented with cough and chest pain and was subsequently diagnosed with metastatic pulmonary adenocarcinoma. The tumor was positive for *Kirsten rat sarcoma virus* oncogene *KRAS*-G12C mutation and had high programmed death-1 ligand expression. She was commenced on first-line chemotherapy with carboplatin and gemcitabine with disease response, then continued on maintenance pemetrexed. She was then commenced on immunotherapy with nivolumab, with complete response for a total of 6 years. She does not report any adverse events. Currently, she shows no evidence of recurrence of non-small cell lung carcinoma.

**Conclusion:**

The exceptional response to immunotherapy seen in this case may be explained by the presence of *Kirsten rat sarcoma virus* oncogene mutation, which is associated with enhanced clinical response to programmed death-1 ligand inhibitors. This report emphasizes the urgent need for further studies evaluating the role of *Kirsten rat sarcoma virus* oncogene mutation in determining the clinical efficacy of immunotherapies. This would enable us to make effective evidence-based clinical interventions in the treatment of non-small cell lung carcinoma.

## Background

Non-small cell lung cancer (NSCLC) accounts for 85% of all lung cancer cases worldwide [1]. The emergence of immunotherapy has transformed the therapeutic landscape of NSCLC. Immune checkpoint inhibitors (ICIs) directed against programmed death-1 ligand (PD-L1), programmed death-1 (PD-1), and cytotoxic T lymphocyte-associated 4 (CTLA-4) proteins are associated with decreased mortality as well as longer progression-free survival in NSCLC patients [2, 3]. Since their introduction in 2015, the role of PD-1 inhibitors has shown tremendous growth, from ancillary treatment to first-line therapies in NSCLC. Pembrolizumab, an antibody against PD-1 receptor, is currently indicated in both first- and second-line therapy in advanced cases of NSCLC without driver mutation, with greater benefit for those with high PD-L1 expression [4]. Nivolumab (anti-PD-1) and atezolizumab (anti-PD-L1) are two other immune-modulating agents recommended as second-line therapy in NSCLC, irrespective of PD-L1 levels [5, 6]. Mutations in the *Kirsten rat sarcoma viral* (*KRAS*) oncogene are one of the most common genetic alterations seen in patients with NSCLC. The *KRAS* gene normally encodes a guanosine triphosphate binding protein that is involved in regulating the activity of the *RAS* gene, which is required for proper growth and development of cells [7]. Mutated *KRAS* results in constitutive activation of the *RAS* gene, leading to unregulated cell growth and development of carcinoma. Patients with *KRAS* mutations generally show decreased response to systemic chemotherapies and were previously associated with poor prognosis [7]. However, there is currently evidence showing increased survival and improved clinical response to ICIs in metastatic NSCLC patients harboring *KRAS* G12C mutation [8]. Sotorasib, which was recently approved for the treatment of NSCLC, is the first agent available against *KRAS* G12C mutation. This calls for further research into the development of novel agents against therapeutic targets in the *KRAS* gene. We report herein a case of advanced NSCLC with *KRAS* mutation and high PD-L1 expression with complete response to immunotherapy after chemotherapy.

## Case presentation

**Fig. 1:**
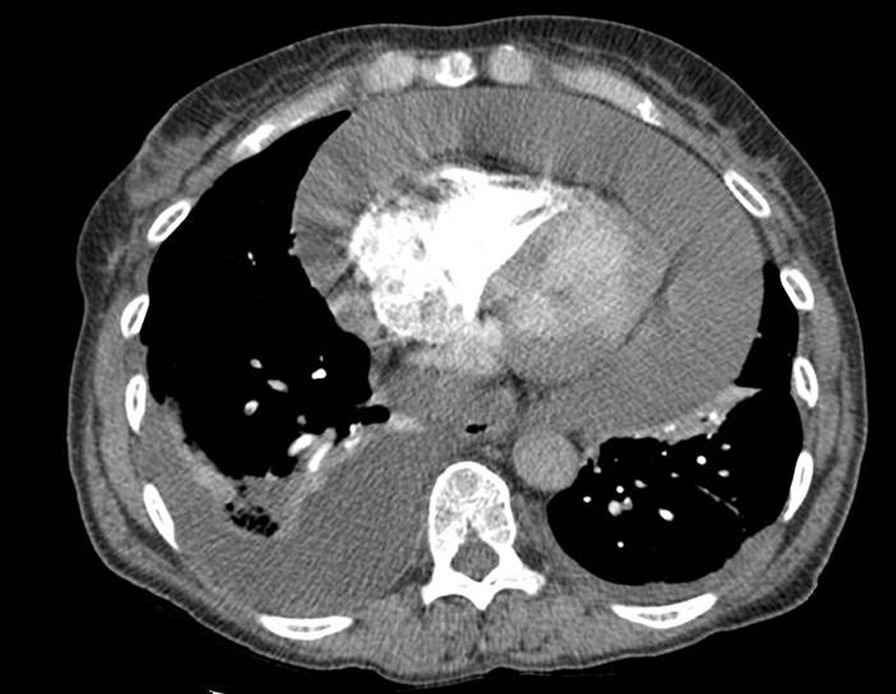
Computed tomography scan showing very large pericardial effusion and possibly reflecting the presence of cardiac tamponade. There is moderate right posterior pleural effusion

A 58-year-old Caucasian female with significant history of smoking and no past medical history presented to the emergency department with chest pain and cough at *T* = 0 in January 2015. Computed tomography (CT) pulmonary angiogram revealed a large pericardial effusion measuring 40 mm in diameter along with moderate right pleural effusion (Fig. 1).

**Fig. 2:**
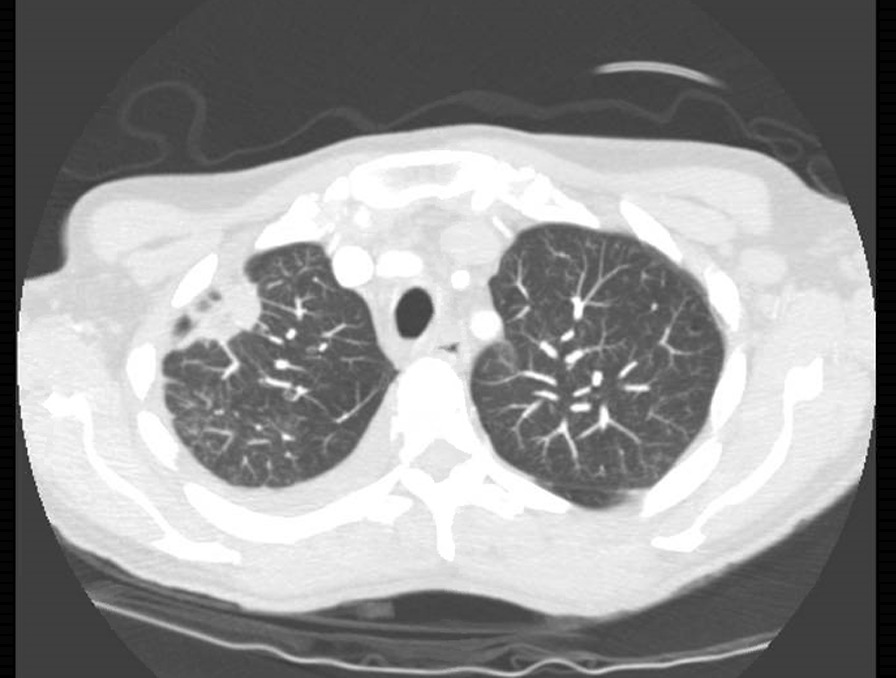
Computed tomography scan showing right upper lobe speculated anterior subpleural lesion extending on the anterolateral pleural surface, suggesting primary bronchial cancer

There was a spiculated anterior subpleural lesion measuring 28 mm extending to the anterolateral pleural surface of the right upper lobe (Fig. 2) and significant enlargement of the mediastinal with right paratracheal lymph node measuring 15 mm and subcarinal lymph node measuring 18 mm and left axillary lymph nodes measuring 12.5 mm. Video-assisted thoracoscopic surgery (VATS) pleurodesis was carried out.

The pericardial and pleural fluid cytology contained small numbers of malignant cells, presenting predominately singly in a bloodstained background. These cells have enlarged pleomorphic nuclei with granular to coarse chromatin, prominent nucleoli, and irregular nuclear contours. Immunohistochemical studies showed positive staining of tumor cells for thyroid transcription factor-1 (TTF-1), napsin A, and Cytokeratin 7 (CK7). Staining for Estrogen receptor (ER), Cytokeratin 20 (CK20), Caudal Type Homeobox 2 (CDX2), and calretinin were all negative. These findings are in support of adenocarcinoma of lung origin.

In 2015, the fluid sample sent was not sufficient to undergo further testing to determine PD-L1 expression, immunohistochemistry testing for  c-ros oncogene 1 (ROS1) / anaplastic lymphoma kinase (*ALK*) mutation, or molecular testing for epidermal growth factor receptor (*EGFR*).

With the imaging and histology findings, she was diagnosed with stage IV (T3N2M1a) according to the TNM classification of the Union of International Cancer Control (UICC), 7th edition lung adenocarcinoma [9]. She had Eastern Cooperative Oncology Group (ECOG) performance status of 1 after VATS pleurodesis. The patient quit smoking at the time of diagnosis. She was then commenced on first-line chemotherapy, carboplatin (AUC 5), and gemcitabine (1000 mg/m^2^) 3 weekly at *T* = 1 month in February 2015. She completed four cycles with no dose reduction. Follow-up CT imaging was done 3 months later (*T* = 4 months) demonstrated good clinical and radiological response. According to the Response Evaluation Criteria in Solid Tumors (RECIST) version 1.1 [10], complete response was achieved with disappearance of the pleural based right upper lobe lesion. There is complete response to the mediastinal, hilar, and axillary lymph nodes. She was clinically well with maintenance of her performance status.

Given the excellent response after first-line chemotherapy, she was then commenced on maintenance chemotherapy with pemetrexed at a dose of 500 mg/m^2^ every 21 days. This is based on evidence that it prolongs overall survival and progression-free survival [11]. The patient completed eight cycles of pemetrexed without significant toxicities.

**Fig. 3:**
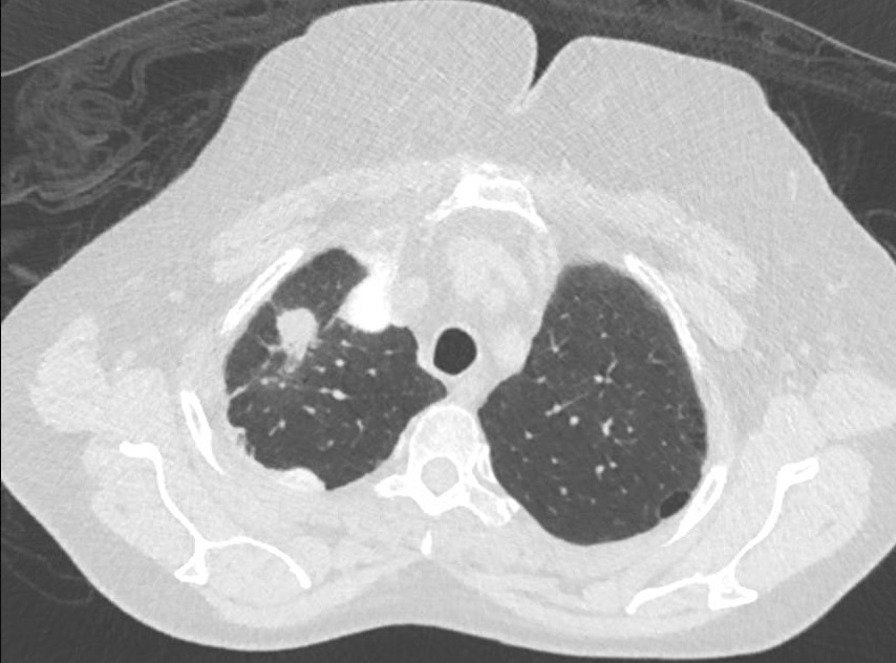
Computed tomography scan showing a right upper lobe lung lesion

In December 2015, there was single lesion progression with a new right upper lobe lesion (18 × 15 mm^2^) (Fig. 3). The patient declined biopsy of this lesion. Given disease progression as per RECIST version 1.1, she was commenced on nivolumab 240 mg every 2 weeks at around *T* = 12 months in February 2016 owing to emerging evidence of immunotherapy in this setting for previously treated metastatic non-small cell lung cancer.

After the commencement of immunotherapy, her follow-up scan done in May 2016 showed complete response of single lung lesion as per RECIST version 1.1. Further imaging subsequently every 3 months with CT of brain, chest, abdomen and pelvis continues to show no evidence of recurrence or new metastatic disease.

At *T* = 59 months in November 2019, she had an elective cholecystectomy for chronic cholecystitis secondary to multiple cholelithiases. Her nivolumab was not withheld during the surgery, and she did not develop any complications during the perioperative period.

At *T* = 63 months in March 2020, somatic gene mutation analysis was requested on her previous pericardial fluid and tissue sample done early in the diagnosis as the patient was not keen for rebiopsy. Reanalysis of the sample could be done using molecular testing. A mutation was detected in the *KRAS* gene p.(Gly12Cys): G12C (16%). No clinically relevant mutation was found in the tested regions of *EGFR*, *BRAF*, *MET*, *RET*, or *ERBB2*. It was negative for rearrangements in *ALK* and *ROS1* genes. There was high level PD-L1 expression with a tumor proportion score (TPS) of 80%.

**Fig. 4:**
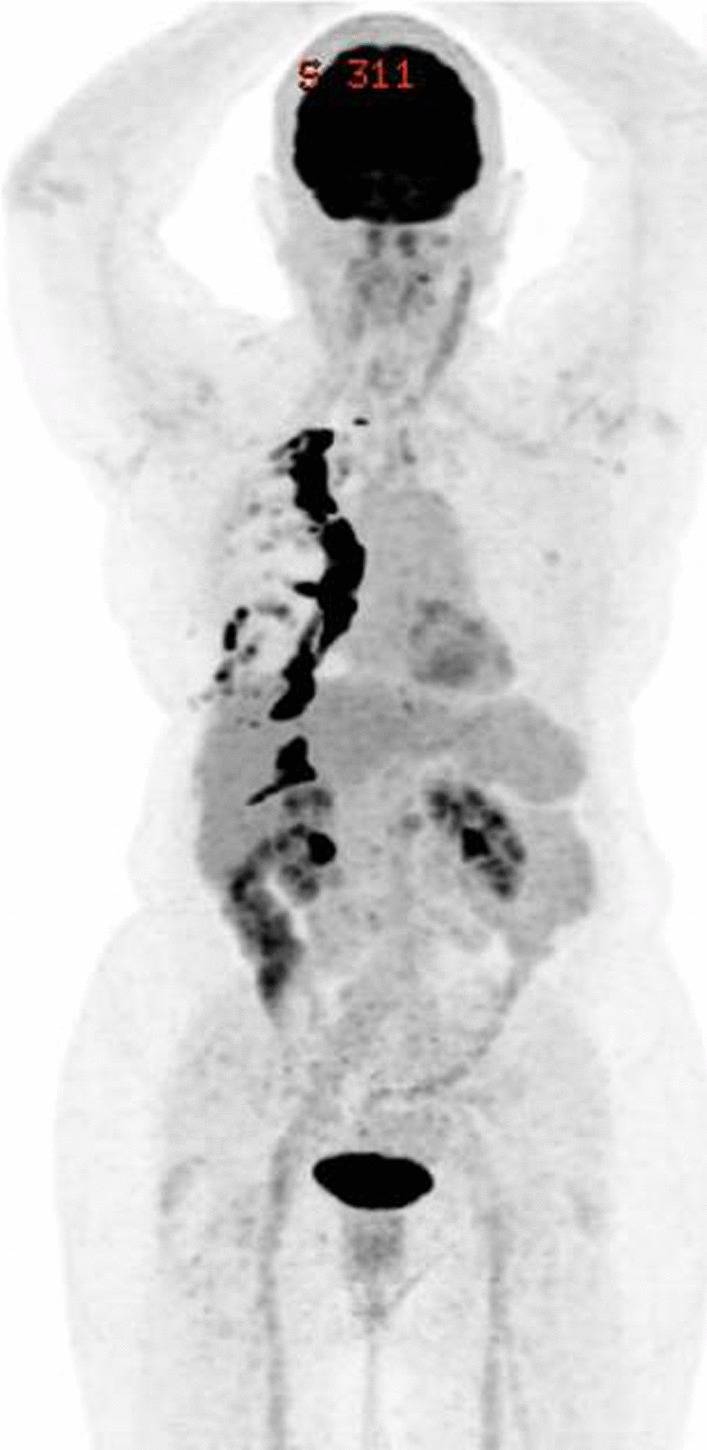
FDG-PET scan showing no suspicious FDG uptake within lungs. There is no scan evidence of pericardial effusion. The foci of intense FDG uptake surrounding right lung pleura are consistent with a previous talc pleurodesis

18-Fluorodeoxyglucose positron emission tomography (FDG-PET) scan at *T* = 83 months in November 2021 showed no suspicious fluorodeoxyglucose (FDG) uptake within lungs. The foci of intense FDG uptake surrounding right lung pleura is consistent with a previous talc pleurodesis (Fig. 4). The patient is currently continuing her immunotherapy and has no evidence of lung malignancy.

## Discussion and conclusions

The advent of immunotherapies has brought about a monumental change in the prognosis as well as management of NSCLC. Therapies with ICIs are associated with improved patient survival, better tolerance, and reduced adverse effects. Although immunotherapies have tremendously enhanced patient outcomes in NSCLC, little is known about its predictive biomarkers and their efficacy in cancers with driver gene mutations. In this report, we describe a case of advanced NSCLC with *KRAS* mutation which has shown outstanding response to immunomodulators and in which the patient has been in complete response since she was started on nivolumab 6 years back.

Although ICIs are associated with improved clinical outcomes, studies assessing the efficacy of nivolumab in pretreated patients with advanced NSCLC reported a median overall survival of 11 months and progression-free survival of 2.5 months as per the 5-year outcome of the Checkmate 017 and Checkmate 057 landmark trials [12]. While ICIs are associated with long-term clinical benefits, most patients develop resistance to these agents within 1–2 years [6]. However, we report a rare case in which the patient has shown excellent response to nivolumab with complete and durable response for the past 6 years, with no signs of resistance. This may be partly explained by the presence of *KRAS* mutation. *KRAS* mutation is seen in 25–30% of patients with NSCLC in the Western world, with the majority of them occurring in codon 12. The common *KRAS* mutations include G12C (43%), G12V (18%), and G12D (11%) [13]. *KRAS* mutation is also strongly associated with smoking history, and only 5% of *KRAS* mutations are seen in nonsmokers [7]. Moreover, G12C and G12V are more common among smokers, compared with G12D in nonsmokers [13]. *KRAS* mutation is associated with enhanced expression of PD-L1 ligand in NSCLC owing to stimulation of downstream pathways [14, 15]. PD-L1 expression also depends on environmental factors. *KRAS* mutant lung cancers in smokers are reported to have greater PD-L1 expression compared with those in nonsmokers [16]. There is compelling evidence to suggest that *KRAS* mutation can result in longer progression-free survival, greater clinical benefits, and improved survival with anti-PD-1 treatment compared with wild-type patients [15, 17]. Liu *et al*. also reported that the clinical benefit of monotherapy with anti-PD-1 agents is comparable to that of combination therapy with docetaxel in *KRAS* mutated individuals [15]. In May 2021, the Food and Drug Administration (FDA) approved the use of sotorasib, the first-ever KRAS inhibitor to be used in targeted therapy. It was approved as a first-line agent in NSCLC patients with G12C mutation who have received at least one line of previous systemic therapy based on phase II data from the CodeBreaK100 study [18].

It is hypothesized that mitochondrial function can play a role in determining the clinical response of anti-PD-1 agents [19]. Factors such as smoking, advanced age, and obesity are associated with impaired mitochondrial function and greater expression of PD-1, resulting in improved clinical response [19]. This can explain the impressive response to immunotherapy for this patient, who was a heavy smoker.

Although immunotherapy has ushered in a new era in cancer therapy, it is not without its limitations. It has been proven that many patients derive significant clinical benefit from immunomodulators, but a large section of patients show no or dismal response to immunotherapies (primary resistance). Low tumor mutational burden (TMB) and expression of heterogeneous antigens leading to decreased tumor immunogenicity, alternate immune checkpoints, and release of immunosuppressive cells such as myeloid-derived suppressive cells and M2 macrophages into the tumor environment are some mechanisms that could contribute to immunotherapy resistance [19]. Host factors such as diet, microbiome, and autoimmunity also contribute to the development of resistance [19]. Aberrations in the interferon-gamma (IFN-γ) signaling pathway leading to defective antigen presentation are now understood as a mechanism of acquired resistance [20]. Even in large clinical trials, the overall response rate was between 47% and 63% [21]. Moreover, the development of acquired resistance after 1–2 years of treatment is also seen. ICIs are also associated with adverse effects, though to a much lesser extent compared withto conventional chemotherapies. Grade 3 or higher adverse effects are seen in 7–13% of total patients treated with PD-1 inhibitors [21]. Serious side effects associated with PD-1 inhibitors include myocarditis, pneumonitis, autoimmune hepatitis, encephalitis, and hypothyroidism [1, 21–25]. PD-L1 assay, though FDA approved, has reduced sensitivity and specificity compared with other diagnostic assays and has limited utility in predicting the clinical benefits of ICI [26]. Hyperprogression of disease is another uncommon adverse effect of anti-PD-1 and PD-L1 therapies, which is characterized by rapid tumor growth or accelerated disease progression once the patient is started on ICI. A meta-analysis by Park *et al*. evaluated the incidence of hyperprogression among patients receiving ICIs and reported a pooled incidence of 13.4% [27].

Herein we present the case of a 58-year-old female with advanced NSCLC who has shown exceptional response to nivolumab therapy after chemotherapy. Given her good response, we plan to continue her treatment until she has disease progression or develops significant toxicities. This decision is supported by the conclusion from the Checkmate 153 trial that overall survival was significantly longer in the continuous treatment with nivolumab arm versus the 1-year fixed-duration arm with retreatment on progression [28].

ICIs are no doubt a landmark development in oncology with the potential to revolutionize the management of lung cancer in the coming years. However, further studies assessing their efficacy should be carried out before reaching definitive conclusions regarding their clinical utility. Targeted therapies against *KRAS* must be given special emphasis because of the tremendous benefit shown in patients with NSCLC. Future research should also focus on the discovery of novel therapeutic targets, newer immunomodulators, and better biomarkers for predicting the clinical benefits of immunotherapies.

## Data Availability

The datasets used and/or analyzed during the current study are available from the corresponding author on reasonable request.

## Abbreviations

NSCLC: Non-small cell lung cancer
ICI: Immune checkpoint inhibitors
PD-L1: Programmed death-1 ligand
PD-1: Programmed death-1
CTLA-4: Cytotoxic T lymphocyte-associated 4
*KRAS*: *Kirsten rat sarcoma virus*
CT: Computed tomography
VATS: Video-assisted thoracoscopic surgery
PET: Positron emission tomography
TPS: Tumor proportion score
TMB: Tumor mutational burden
